# Analysis of the causes and influencing factors of fetal loss in advanced maternal age: a nested case-control study

**DOI:** 10.1186/s12884-021-04027-6

**Published:** 2021-08-04

**Authors:** Xiaomei Wang, Yuan Lin, Zhaozhen Liu, Xinxin Huang, Rongxin Chen, Huihui Huang

**Affiliations:** 1 Department of Obstetrics, Fujian Maternity and Child Health Hospital, Affiliated Hospital of Fujian Medical University, No. 18 Daoshan Road, Gulou District, Fuzhou, Fujian 350001 People’s Republic of China; 2 Department of Gynecology, Fujian Maternity and Child Health Hospital, Affiliated Hospital of Fujian Medical University, No. 18 Daoshan Road, Gulou District, Fuzhou, Fujian 350001 People’s Republic of China; 3 Healthcare Department, Fujian Maternity and Child Health Hospital, Affiliated Hospital of Fujian Medical University, No. 18 Daoshan Road, Gulou District, Fuzhou, Fujian 350001 People’s Republic of China

**Keywords:** Maternal age, Spontaneous abortion, Stillbirth, Congenital abnormality, Nested case-control study

## Abstract

**Background:**

The risk of fetal loss is higher among ≥35-year-olds than younger women. The present study aimed to explore the causes and factors influencing fetal loss in advanced maternal age (AMA).

**Methods:**

AMA women with singleton fetuses (< 14 gestational weeks) who underwent their first prenatal examination in the Obstetrics Department of Fujian Maternity and Child Health Hospital from December 2018 to June 2020 were included in this cohort study. Those who terminated the pregnancy before 14 gestational weeks were excluded. A baseline survey was conducted, and follow-up was carried out until the termination of the pregnancy. Clinical data were extracted to analyse the causes of fetal loss among them. In the nested case-control study, the AMA women with fetal loss were enrolled as the case group, and women without fetal loss in the same period were enrolled as the control group, in a 1:2 ratio matched by age and gestational weeks. Logistic regression models were used to analyse the factors influencing fetal loss.

**Results:**

A total of 239 women with fetal loss and 478 controls were enrolled. The causes of fetal loss were most often fetal factors, followed by maternal factors, umbilical cord factors, and placental factors. Multivariate logistic regression analysis indicated that junior high school education and below (adjusted odds ratio (aOR) = 5.13, 95% confidence interval (CI): 2.19–12.02), senior high school education (aOR = 4.91, 95% CI: 2.09–11.54), residence in a rural area (aOR = 2.85, 95% CI: 1.92–4.25), unemployment (aOR = 1.81, 95% CI: 1.20–2.71), spontaneous abortion history (aOR = 1.88, 95% CI: 1.26–2.80), preterm birth history (aOR = 11.08, 95% CI: 2.90–42.26), hypertensive disorders of pregnancy (aOR = 7.20, 95% CI: 2.24–23.12), and preterm premature rupture of membranes (aOR = 4.12, 95% CI: 1.53–11.11) were risk factors for fetal loss.

**Conclusions:**

Low educational level, unemployment, abnormal pregnancy/labor history, and pregnancy complications were correlated with the incidence of fetal loss in AMA. Thus, early identification as well as a targeted intervention, should be conducted.

## Background

An increasing trend in maternal childbearing age has been observed worldwide [1]. The increase in average pregnancy age could be attributed to the increase in the number of women aged ≥35 years attempting to conceive. Advanced maternal age (AMA) is defined as a maternal age of ≥35 years at the expected delivery time [2]. In the USA, the birth rates among AMA women increased by 12% from 2007 to 2016 [3]. Interestingly, AMA has been associated with high academic and career pursuit, delayed conception due to infertility, and prolonged life expectancy [4]. Zhang et al. [5] found that the prevalence of AMA was 15.82% in 2017 in Zhejiang Province, China.

AMA has been associated with adverse pregnancy outcomes, including spontaneous abortion, fetal chromosomal and congenital abnormalities, spontaneous late preterm delivery, and stillbirth [6–8]. Abortion, stillbirth, or induced labor can lead to fetal loss [9]. Some studies have shown that the risk of adverse perinatal outcomes is elevated with increasing maternal age [10].

However, these observational studies focused on the correlation between maternal age and adverse perinatal outcomes, and most conclusions were derived from studies in which women with AMA were compared to those < 35 years old. Other clinical indicators associated with adverse birth outcomes in AMA were not studied comprehensively, necessitating further investigation of other risk factors correlated with adverse birth outcomes in AMA. In addition, the causes of fetal loss can be divided into maternal, fetal, placental, and cord factors, while only a few studies have focused on AMA.

Although complicated, it is valuable to identify the causes and factors influencing fetal loss in AMA. Early abortion is partially an unintended pregnancy. Thus, based on a cohort of AMA women, this nested case-control study explored the causes and risk factors for fetal loss during the second and third trimesters in AMA women to provide a theoretical basis for the effective prevention and control of fetal loss at AMA.

## Methods

### Subjects

AMA women with singleton fetuses (< 14 gestational weeks) who underwent their first prenatal examination in the Obstetrics Department of Fujian Maternity and Child Health Hospital from December 2018 to June 2020 were included in this cohort study. Those who terminated the pregnancy before 14 gestational weeks were excluded. The baseline survey and follow-up until termination of pregnancy were conducted for all participants.

In this nested case-control study, AMA women with fetal loss at 14 weeks of gestation or later were enrolled as the case group; fetal loss included induced labor due to fetal abnormality, spontaneous or artificial abortion, and stillbirth [9]. The fetal abnormalities included congenital abnormalities and chromosomal abnormalities, such as anencephalus, single atrium, single ventricle, gastroschisis, thanatophoric dwarfism, trisomies, and monosomy X [11]. In China, abortion refers to spontaneous or artificial abortion < 28 weeks of pregnancy. The causes of artificial abortion were severe pregnancy complications, such as hypertensive disorders of pregnancy (HDP), preterm premature rupture of membranes (PPROM), and intra-amniotic infection. Stillbirth was defined as an intrauterine fetal death at a gestational age of ≥20 weeks, including intrapartum stillbirth after the onset of labor but before birth [12]. The participants with unintended termination of pregnancy and the survival of extremely premature infants (< 28 gestational weeks) were excluded from the study.

Women without fetal loss in the same period constituted the control group. They were enrolled in the nested case-control study at a 1:2 ratio with the case group, matched by age and gestational weeks. They were followed up according to regular antenatal care for pregnancy outcomes of full-term live birth. If fetal loss occurred during the follow-up, the patient was switched to the case group. The inclusion criteria for the control group were as follows: the age difference between the control and case subjects < 1 year and difference in gestational weeks between the two groups < 2 weeks.

### Research methods

Baseline data were collected by questionnaire, medical history inquiry, physical examination, and laboratory and ultrasound examination. Follow-up was conducted according to the timing of the regular antenatal examinations, and the information from each antenatal examination until termination of pregnancy was recorded. The questionnaire survey included paternal age and demographic characteristics of the pregnant women, such as maternal age, race, education, career, residential quarter, and pre-pregnancy body mass index (BMI).

The causes of fetal loss were divided into maternal, fetal, placental, and cord factors, though some causes of death may be unknown. Maternal factors included pregnancy complications. Fetal factors included congenital fetal abnormalities, chromosomal abnormalities, and fetal oedema. When a fetal chromosomal abnormality was accompanied by congenital malformation, it was considered a chromosomal abnormality. Placental factors included placental abruption, histological chorioamnionitis, and placental infarction, and umbilical cord factors included cord torsion, cord entanglement, and prolapse of the umbilical cord [11]. When there were multiple causes of fetal loss, the main cause was counted. Baseline data, the information from each antenatal examination, fetal chromosomal karyotype, ultrasonic examination, magnetic resonance imaging (MRI), and placental pathological reports of fetal loss cases were extracted to analyse the causes of fetal loss.

Variables such as demographic characteristics, pregnancy/labor history, and pregnancy complications were collected from the case and control groups to analyse fetal loss factors. The demographic characteristics included maternal education, residential area, career, and pre-pregnancy body mass index. The history of pregnancy labor included gravidity, previous parity, spontaneous abortion history, preterm birth history, stillbirth history, and congenital malformation history. The pregnancy complications of the two groups were collected at enrolment, including diabetes mellitus (pregestational and gestational), HDP, fetal growth restriction, PPROM, placenta previa, velamentous cord insertion, torsion of cord, and in vitro fertilization and embryo transfer (IVF-ET). The diagnostic criteria for pregnancy complications and pregnancy outcomes were defined according to the relevant guidelines.

### Statistical analysis

The data were organized in Excel (Microsoft Co., Redmond, WA, USA), and all statistical analyses were performed using SPSS 25.0 software (IBM Co., Armonk, NY, USA). The measurement data were tested for normality. If the distribution of continuous variables was normal, the data are presented as mean ± standard deviation (SD), while if the distribution was non-normal, the data are presented as median and interquartile range. Categorical variables are expressed as numbers and percentages.

Univariate and multivariate conditional logistic regression models were used to analyse the influencing factors associated with fetal loss. The variables with statistical significance in univariate analysis were included in the multivariate logistic regression analysis (backward). In SPSS, the value of slentry was 0.05, and slstay was 0.15. Odds ratio (*OR*) and 95% confidence interval (95% CI) were used to report the effect estimates. All tests were two-sided. A *P*-value < 0.05 was considered statistically significant.

## Results

### General information

**Fig. 1:**
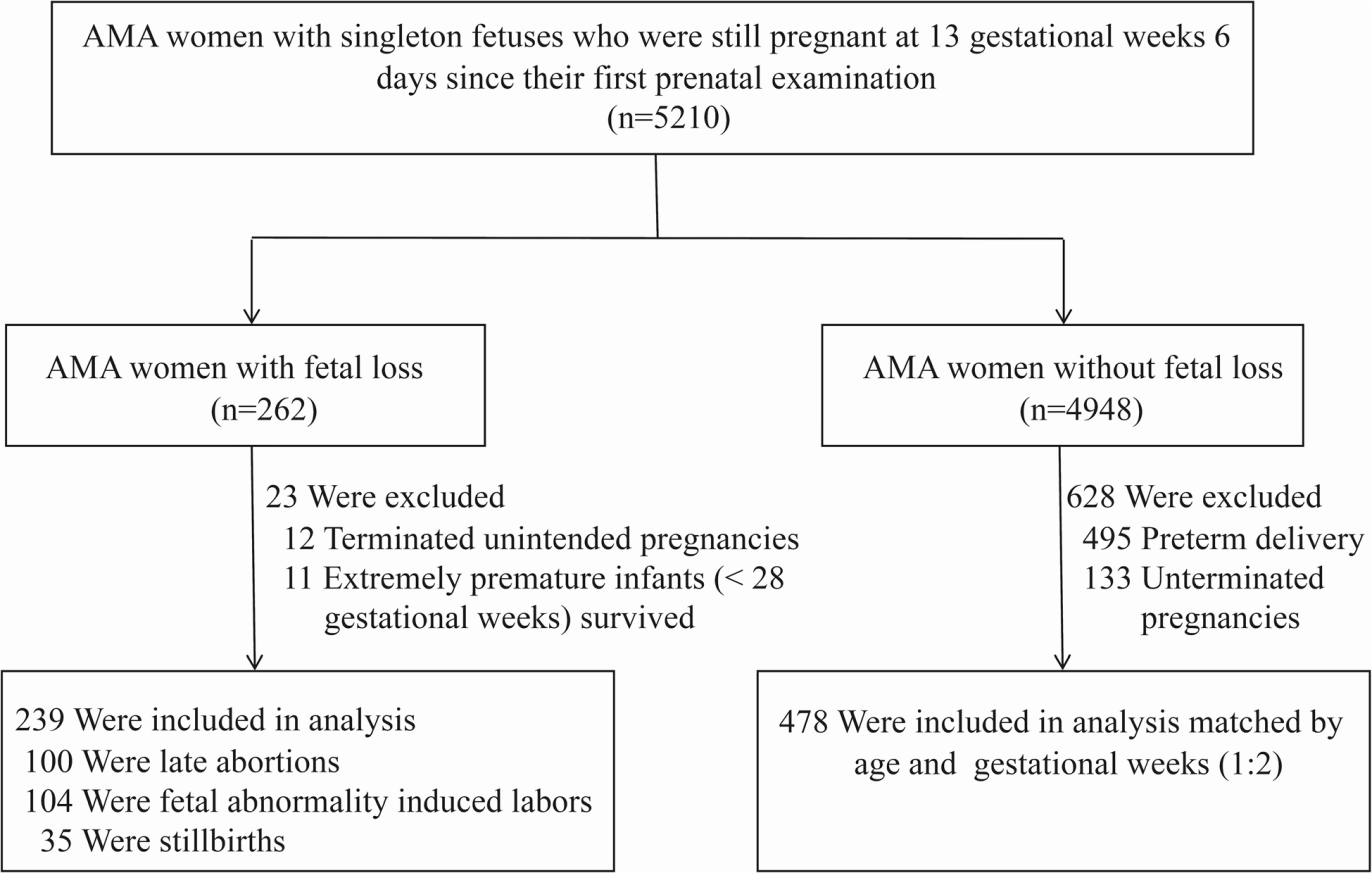
The process for selecting participants

From December 2018 to June 2020, 5210 AMA women with singleton fetuses who were still pregnant at 13 gestational weeks 6 days since their first prenatal examination, were enrolled in this AMA cohort study. In the cohort, 239 cases of fetal loss were eligible for the case group. The gestational age at fetal loss was between 14 and 39 weeks, and 47 cases occurred in the third trimester. The process for selecting participants who were assigned to the case and control groups is presented in Fig. 1. There were no significant differences between the two groups in maternal age, race, or paternal age (Table 1).

**Table 1:** General characteristics of the participants

Variable	Fetal Loss Group (***n*** = 239)	Control Group (***n*** = 478)	***p*** value
Maternal age at expected date of delivery-yr			
Mean ± SD	37.45 ± 2.57	37.47 ± 2.53	0.90
Paternal age-yr			
Mean ± SD	39.15 ± 4.18	39.22 ± 3.33	0.84
Race-no. (%)			0.90
Han	237 (99.16)	472 (98.74)	
Other	2 (0.84)	6 (1.26)	

### Causes of fetal loss

The causes of fetal loss were most often fetal factors, followed by maternal factors, umbilical cord factors, and placental factors. However, because genetic chromosomal tests and autopsies were not performed in every fetal loss case, some fetal loss causes were unknown (Table 2). Among the fetal factors, according to ultrasonic examination, MRI, fetal chromosomal karyotype, and fetal cadaver appearance examination, congenital abnormalities accounted for 49.04% (51/104), chromosomal abnormalities 46.15% (48/104), and oedema 4.81% (5/104). Congenital abnormalities primarily included heart, central nervous system, skeletal, and muscular malformations. The chromosomal abnormalities were mainly trisomy 21, 18, and Klinefelter syndrome. The top four maternal factors were as follows: 17 cases of severe preeclampsia (PE), 17 cases of intra-amniotic infection, 11 cases of PPROM (between 20 and 25 gestational weeks), and 5 cases of cervical insufficiency. Placental factors included 14 cases of histological chorioamnionitis and 6 cases of placental abruption. The umbilical cord factors were torsion of the cord, cord entanglement, excessively short cord, and prolapse of the cord; 36.36% (8/22) of these happened in the third trimester. Unknown causes of fetal loss were seen mainly at 14–19 weeks (73.53%, 25/34).

**Table 2:** Gestational age distribution of causes of fetal loss in second and third trimester

Causes	Second trimester (***n*** = 192)			Third trimester (***n*** = 47)			Total, n (%)
	14–19^**+ 6**^ (***n*** = 58)	20–23^**+ 6**^ (***n*** = 62)	24–27^**+ 6**^ (***n*** = 72)	28–31^**+ 6**^ (***n*** = 26)	32–36^**+ 6**^ (***n*** = 19)	≥ 37 (***n*** = 2)	
Fetal factors	14 (24.14)	27 (43.55)	44 (61.11)	12 (46.15)	7 (36.84)	0 (0.00)	104 (43.51)
Maternal factors	13 (22.41)	18 (29.03)	12 (16.67)	9 (34.62)	6 (31.58)	1 (50.00)	59 (24.69)
Umbilical cord factors	1 (1.72)	6 (9.68)	7 (9.72)	3 (11.54)	4 (21.05)	1 (50.00)	22 (9.21)
Placental factors	5 (8.62)	6 (9.68)	5 (6.94)	2 (7.69)	2 (10.53)	0 (0.00)	20 (8.37)
Unknown reasons	25 (43.10)	5 (8.06)	4 (5.56)	0 (0.00)	0 (0.00)	0 (0.00)	34 (14.23)

### Influencing factors of fetal loss

Statistically significant variables in the univariate conditional logistic regression were education status, residence, employment, prepregnancy BMI, previous parity, spontaneous abortion history, preterm birth history, HDP, PPROM, velamentous cord insertion, and IVF-ET (Table 3). Multivariate analysis showed that compared with postgraduate education or above, junior high school and below (aOR = 5.13, 95% CI: 2.19–12.02) and senior high school education (aOR = 4.91, 95% CI: 2.09–11.54) were risk factors for fetal loss. Residence in a rural area (aOR = 2.85, 95% CI: 1.92–4.25), unemployment (aOR = 1.81, 95% CI: 1.20–2.71), spontaneous abortion history (aOR = 1.88, 95% CI: 1.26–2.80), preterm birth history (aOR = 11.08, 95% CI: 2.90–42.26), HDP (aOR = 7.20, 95% CI: 2.24–23.12), and PPROM (aOR = 4.12, 95% CI: 1.53–11.11) increased the risk of fetal loss (Table 4).

**Table 3:** Analysis results on influencing factors of fetal loss by univariate conditional logistic regression model

Influencing factor	Case group (***n*** = 239)	Control group (***n*** = 478)	***OR*** value	95% CI
Education status				
Postgraduate or above	11 (4.60)	69 (14.44)	1.00	–
Junior high school and below	63 (26.36)	35 (7.32)	11.29	5.29–24.11
Senior high school	39 (16.32)	34 (7.11)	7.20	3.28–15.78
Undergraduate	126 (52.72)	340 (71.13)	2.33	1.19–4.54
Residence in a rural area	108 (45.19)	81 (16.95)	4.03	2.84–5.72
Unemployment	110 (46.03)	100 (20.92)	3.22	2.30–4.51
Pre-pregnancy BMI^a^				
18.5–23.9	167 (69.87)	381 (79.71)	1.00	–
< 18.5	13 (5.44)	30 (6.28)	5.60	1.88–16.75
24.0–27.9	42 (17.57)	60 (12.55)	3.47	1.32–9.10
≥ 28.0	17 (7.11)	7 (1.46)	5.54	2.26–13.61
Gravidity				
≥ 3	152 (63.60)	297 (62.13)	1.00	–
1	15 (6.28)	35 (7.32)	0.84	0.44–1.58
2	72 (30.13)	146 (30.54)	0.96	0.68–1.36
Previous parity				
≥ 2	37 (15.48)	40 (8.37)	1.00	–
0	39 (16.32)	60 (12.55)	0.70	0.39–1.28
1	163 (68.20)	378 (79.08)	0.47	0.29–0.76
Preterm birth history	13 (5.44)	3 (0.63)	9.15	2.58–32.43
Spontaneous abortion history	75 (31.38)	101 (21.13)	1.71	1.20–2.42
Stillbirth history	2 (0.84)	2 (0.42)	0.50	0.07–3.54
Congenital malformations history	10 (4.18)	9 (1.88)	0.44	0.18–1.09
Hypertensive disorders of pregnancy	22 (9.21)	4 (0.84)	12.01	4.09–35.29
Preterm premature rupture of membranes	14 (5.86)	8 (1.67)	3.66	1.51–8.84
Velamentous cord insertion	7 (2.93)	3 (0.63)	4.78	1.22–18.64
In vitro fertilization and embryo transfer	22 (9.21)	24 (5.02)	1.92	1.05–3.50
Pregestational diabetes mellitus	5 (2.09)	4 (0.84)	2.53	0.67–9.52
Gestational diabetes mellitus	23 (9.62)	37 (7.74)	1.27	0.74–2.19
Fetal growth restriction	1 (0.42)	1 (0.21)	2.00	0.13–32.18
Placenta previa	4 (1.67)	7 (1.46)	1.15	0.33–3.95
Torsion of cord	18 (7.53)	21 (4.39)	1.77	0.93–3.39

^a^The body-mass index (BMI) is the weight in kilograms divided by the square of the height in meters

**Table 4:** Analysis results on influencing factors of fetal loss by multivariate conditional logistic regression model

Influencing factor	***aOR*** value	95% CI
Education status		
Junior high school and below	5.13	2.19–12.02
Senior high school	4.91	2.09–11.54
Undergraduate course	1.93	0.94–3.94
Residence in a rural area	2.85	1.92–4.25
Unemployment	1.81	1.20–2.71
Spontaneous abortion history	1.88	1.26–2.80
Preterm birth history	11.08	2.90–42.26
Hypertensive disorders of pregnancy	7.20	2.24–23.12
Preterm premature rupture of membranes	4.12	1.53–11.11
Velamentous cord insertion	4.61	0.99–21.44
In vitro fertilization and embryo transfer	1.78	0.89–3.54

## Discussion

The advancements in assisted reproductive technology and the two-child policy in China have increased the proportion of AMA, and fetal loss has increased accordingly. Fetal loss has a negative impact on maternal physical and mental health in the case of AMA. Therefore, we needed to find appropriate and timely interventions to reduce fetal loss in AMA women.

### Aetiology and countermeasures against fetal loss

This study found that fetal factors were the primary cause of fetal loss in AMA, as found in the study by Walker et al. [8]. Congenital malformations, especially heart malformations [13], cause the majority of the fetal losses. Therefore, fetal systemic color Doppler ultrasonography and echocardiography are performed between 18 and 24 gestational weeks, assisted by MRI when necessary, to increase the detection of malformations [14]. In addition, genetic technologies including chromosomal microarray analysis, whole-exome and whole-genome sequencing, have improved prenatal diagnosis [15]. Upon the diagnosis of a fetal abnormality, the treatment should be based on gestational age, severity, and prognosis. A multidisciplinary team should provide genetic counselling, allowing patient to make an informed choice at the earliest about whether to continue the pregnancy, thereby avoiding severe fetal abnormality in the perinatal period. In recent years, fetal surgery has been attempted in cases of severe hydrocephalus and diaphragmatic hernia [16].

Among maternal factors, HDP, especially severe PE, could result in serious perinatal problems [17]. A total of 17 cases of severe PE resulted in a fetal loss in the present study. The treatment includes reducing blood pressure when indicated, prevention of convulsions, close monitoring of maternal and fetal conditions, prevention and treatment of complications, and timely termination of the pregnancy to reduce adverse outcomes [18]. Infection is the most common complication of PPROM. However, if maternal and fetal fitness are optimal between 24 and 27 weeks of gestation, expectant management reduces fetal loss. It consists of ultrasonographic monitoring of fetal growth, amniotic fluid, and fetal heart rate while assessing the presence of fetal abnormalities, clinical amniotic cavity infection, and significant placental abruption as indications of delivery [19].

Umbilical cord factors often lead to fetal distress and stillbirth, and thus are deemed a significant cause of fetal loss in the third trimester. Pathological examination of the placenta is also valuable, but this might be done too late to prevent fetal loss. Therefore, it is necessary to improve the prenatal diagnosis of umbilical cord entanglement, the helix index, umbilical cord position, and insertion by color Doppler ultrasound. In addition, timely reporting and appropriate management might prevent stillbirth in the third trimester if reduced fetal movement is found [20]. Fetal loss by unknown causes mainly occurred between 14 and 19 weeks of gestation in this study, which might be related to the performance of systemic color Doppler ultrasonography at non-optimal times and the low autopsy rate at this stage.

### Correlations between the influencing factors and fetal loss

The multivariate logistic regression analysis in this study confirmed that a low educational level was a risk factor for fetal loss, as described previously by Zhu et al. [12], The lower the level of education, the higher the risk. Additionally, an increased risk of fetal loss was noted in unemployed women and in women living in rural areas, which may be associated with low socioeconomic status [21]. Antenatal care for these women should be strengthened to prevent ignorance of symptoms related to pregnancy complications that could threaten maternal and infant health. In China, BMI is categorized as follows: underweight (< 18.5 kg/m^2^), normal weight (18.5–23.9 kg/m^2^), overweight (24–27.9 kg/m^2^), and obesity (≥ 28 kg/m^2^) [22]. Yi et al. [23] indicated that pre-pregnancy obesity was associated with a higher risk of stillbirth than normal weight; thus, obese pregnant women should be guided towards an appropriate diet and exercise. The univariate analysis in this study showed that pre-pregnancy overweight and obesity were associated with fetal loss.

We also found that women with previous spontaneous abortion or preterm birth had an increased risk of fetal loss. A population-based study showed that stillbirth was highly prevalent among women with previous spontaneous abortion or stillbirth [24]. Moreover, HDP and PPROM increased the risk of fetal loss, and the risk of antepartum stillbirth in hypertensive women showes a positive correlation with disease severity [25]. The average paternal age in this study was 39 years. A previous study showed that advanced paternal age was correlated with stillbirth and congenital malformations, which could be attributed to the loss of sperm DNA integrity or an increase in the incidence of de novo mutations [26]. IVF-ET has been correlated with a high risk of stillbirth and congenital malformations in ART singleton infants [27, 28]. Thus, future studies could explore the effect of paternal age and assisted reproduction on fetal loss.

The current trial has some limitations. First, this was a single-centre study limited to specialized tertiary hospitals. Second, nutritional status during pregnancy, vitamin intake, paternal health, and other factors were not investigated. Due to the limitation of sample size and low incidence rates, we did not include data on maternal diseases involving the kidney, thyroid, or immune diseases.

## Conclusions

In summary, low educational level, unemployment, abnormal pregnancy/labor history, and pregnancy complications were correlated with fetal loss in AMA women. Early identification of risk factors, targeted health education, enhanced evaluation of complications, and better antenatal care should be given high importance to reduce fetal loss caused by maternal factors and avoid severe fetal abnormalities in the perinatal period. Furthermore, it is necessary to improve the prenatal diagnosis of umbilical cord abnormalities and strengthen fetal monitoring in the third trimester.

## Data Availability

All data generated or analysed during this study are included in this published article.

## Abbreviations

AMA: Advanced maternal age
HDP: Hypertensive disorders of pregnancy
PPROM: Preterm premature rupture of membranes
BMI: Body mass index
IVF-ET: In vitro fertilization and embryo transfer
PE: Preeclampsia
MRI: Magnetic resonance imaging
